# Round the Hospitals

**Published:** 1918-10-19

**Authors:** 


### ROUND THE HOSPITALS.

The motto for London this week, "It's your money we want," is vociferated in every house, at every street corner. The supreme demands of the State appeal to prudence and patriotism; the irresistible claims of the Red Cross to pity and to that ancient classical *pietas*, or reverence for humanity, which we miscall piety. Talk about millions is apt to leave the ordinary impecunious individual cold, and in few institutions is there that fervour for saving which can be inspired by concerted action. There could be no worthier contribution to the Government appeal this week than to start a War Savings Association in every hospital still unprovided with this stimulus. The weekly shilling which almost every worker could save for the State is a poor thing by itself. But as one of a million others it assumes proportions of dignity and sweeps the country to victory. To capture it week by week from every member of the community is the work of the War Savings Associations, and the task is one of the worthiest of the hour.

---

The Referee has sent us the following brief report of the success of the Leaflet Competition:—

> In all, 53 contributions and papers have been received for the competition announced in our columns during August and September. They are for the most part of high quality and form an eloquent tribute to the love for their profession cherished by nurses working in every branch of it.

We hope to announce the result of the competition with all possible dispatch. It is the intention to give a full report, that the range and value of the leaflets sent in for competition may be readily appraised by our readers. If these leaflets are in demand, arrangements for their publication will be made.

---

The grave shortage of beds for the wounded which has resulted out of the long-sustained and victorious advance of the Allied troops has compelled the War Office to appeal to all the auxiliary hospitals to enlarge their accommodation. In some this is impossible, but the call is being responded to with alacrity in most localities. The total number of auxiliary home hospitals actually at work under the British Red Cross was 1.165 at the end of August, with a total of 67,652 beds. This shows an increase of 2,454 beds during the month. And, in addition, eleven hospitals with a total of 697 beds have been accepted by the Admiralty through the British Red Cross Society. The best of these small hospitals may have a value in developing the organising powers of the community and in training some nursing sisters in the delicate art of matronship. They have done some useful work in war-time, but not a few have left much to desire. They will last their time and have cultivated sympathy and a knowledge of the trials and conditions of our wounded warriors of all Services.

---

The Vice-Chairman of King Edward VII.'s Hospital, Cardiff, has taken the unusual but, as it seems to us, very sensible step of appealing for probationers in the local press. He writes an admirable letter, and expresses the need for both part-time helpers and probationers to give themselves unreservedly to the profession of nursing. The hospital has the great advantage of a well-equipped preliminary training-school at Anthony House, which is under the superintendence of Sister Gould. Nothing acts as a better inducement to young and well-educated women to present themselves for training than the preliminary school, which enables them to prove their vocation before being actually committed to the nurse's life.

---

Miss Pegg had something new to say at the Dundee meeting in connection with the College of Nursing, and she said it well. It related to the length of time it is necessary for nurses to spend *in statu pupillari* if they are keen to master all the branches of their profession. As head of a large and particularly well-appointed training-school, Miss Pegg is in entire sympathy with matrons who prefer training their own nurses throughout all stages, but she believes in affiliation and co-operation, and is willing to surrender personal feeling in order to obtain it. She was on sure ground when she pointed out that, whereas the medical student could qualify in all subjects in the course of five years, a nurse who desired to obtain first-class certificates in general, fever, mental, and children's nursing, in maternity, ear, nose, skin, and ophthalmic work, in massage and electricity, would have to spend from eight to eleven years before her full earning time commenced. It is necessary to emphasise the fact that the barriers to complete qualification with a reasonable period have not been erected in the interests of the nurse or of nursing, but are due to other causes.

---

We understand that probationers at the Great Northern Central Hospital are to have higher salaries. The new rate doubles the salary of the first-year probationers (from £8 to £16), raises the second-year probationers by half as much again (from £12 to £18), third-year probationers by one-fourth (from £16 to £20), and fourth-year probationers by one-sixth (from £24 to £28). This is characteristic of the present movement towards raising salaries, and we confess it appears to us not wholly satisfactory. It may be argued that first-year probationers have always been worse paid in proportion than the rest. But, on the other hand, they can only rank as unskilled labour, and their board and lodging, lectures and training, uniform and laundry cost the institution in these days very little short of £70 a year. Add a trifle for pocket-money, and their services are indisputably well paid.

---

The pay of probationers has been artificially forced up by the great demand at present for unskilled labour. This is a matter altogether apart from the burning question of underpaid trained

**ROUND THE HOSPITALS—(*continued*).**

nurses in institutions. It is true that an improvement in the probationers' salaries influences that of the entire staff by throwing into strong relief the poor prospects of such as elect to remain on as staff nurses and ward sisters, but it is rather a matter of present expediency than of justice. The action of the authorities in the Bath United Hospital, where the sisters' salaries were raised two years ago to £60, advancing by yearly increments to £70, is excellent. This institution has lately raised probationers' salaries from £6, £8, £10, £20 to £15, £18, £21, and £28. The sisters and other permanent members of the staff are entitled to first consideration. When their just claims are met the question of securing a good supply of probationers can be properly dealt with.

The new scale of remuneration recently adopted by the Hertfordshire County Nursing Association marks a real advance in one of the worst-paid branches of the profession. The salaries of midwives and of district and village nurses are graded according to population and length of service, an eminently fair arrangement. Briefly, it may be stated that the minimum salary, that for village nurses commencing work among less than 1,000 inhabitants, amounts, with war bonus and allowances, to £89, a figure which would have been deemed fantastic ten years ago. Midwives are to have from a minimum of £107 to a maximum of £127; Queen's nurses, minimum £117, maximum £157, according to population and length of service. It is a well-considered attempt to remove the inequality in the posts open to nurses and secure to all an adequate allowance for board and lodging.

It is not necessary to conclude that salaries will remain at the Hertfordshire level. The needs of the country are very urgent, and we believe district nurses are but now beginning to come into their own. In the West Riding of Yorkshire so much difficulty has been experienced in getting suitable candidates as school nurses and health visitors that the Health Committee of the County Council is advertising for fully-qualified nurses with C.M.B. certificate at a salary of £120, rising by £5 yearly increments to £150, plus travelling expenses and £5 for uniform. This rate of pay includes war bonus, but it is easy to see that these words are but a stepping-stone in most instances to a permanent improvement.

The resignation of Miss E. M. Byles, matron of Lambeth Infirmary for seventeen years, is a distinct loss to the nursing profession of South London. Since the war her duties have enormously increased because the Army Council took over the Southwark Infirmary, when the patients from the latter were transferred to Lambeth. In addition there have been the troubles due to diminished medical and nursing staffs, which have created much anxiety and work. It was little wonder, therefore, that Miss Byles should have a breakdown, which necessitates her resignation. Miss Byles was appointed matron on November 5, 1901, and during the long intervening years her object has been to minister to the many thousands of patients in the infirmary, and to promote the well-being of the nursing staffs. It is to be hoped that she may speedily recover again to take up the work which for so many years she has performed faithfully and conscientiously.

By the death of Dr. Biernacki, medical superintendent of Plaistow Fever Hospital, another landmark in the history of trained nursing passes away. When Dr. Biernacki began his labours the fever nurse was a mere sick-room attendant, without status and with the poorest prospects. He early formed the opinion that the nursing of infectious diseases required more than common intelligence, and an even more precise training in asepsis than that accorded to the surgical nurse. He promptly put his theories into action, and we believe he was the first to introduce that exact system of training which has now brought the fever branch of nursing into deservedly high repute. The women trained at Plaistow carried the torch far and wide. They demonstrated the power and value of a good education in the nursing of fever patients, and have helped to bring many institutions where the nursing was of a retrograde kind up to a high standard.

As founder and chief organiser of the Fever Nurses' Association Dr. Biernacki did much to ensure that fever nurses should obtain that measure of general training necessary to complete their qualifications. The scheme he formulated, if not ideally perfect, has worked remarkably well, and is likely to be taken as a basis in any reconstruction. Naturally enough, he laid greater stress on the value of the fever part of the training than on that gained in general hospitals, but he was an enthusiast, and was justified by the results he was able to bring about.

An urgent appeal for a voluntary corps of women workers as ward aides in the Middlesex Hospital has been published in a daily paper under the signature of Sybil, Countess of Brassey, and with the approval of the authorities the corps is to carry out those unskilled but essential duties which form part of the routine work of the wards. A rota will be arranged to suit the convenience of the corps, who must be prepared to give four hours a day for two or three days a week to the work. The idea is an excellent one. Similar voluntary corps, on a small scale, have to our knowledge worked exceedingly well in purely military establishments, and matrons of civilian hospitals, who are at present much hampered, like the Middlesex, for lack of help, could not do better than get one of their supporters to organise part-time workers on similar lines.

**ROUND THE HOSPITALS**—(*continued*).

Yorkshire has always had a fine reputation for open-handedness, and the efforts recently made at Sheffield and Leeds on behalf of the College of Nursing were worthy of it. At Sheffield a sale of work for the benefit of the Local Centre of the College has yielded £320, a very satisfactory nucleus to go to work on. On the initiative of Miss Lindall, of the Hospital for Women and Children, at Leeds, a Flag Day has been held in that city for the benefit of the Nation's Fund for Nurses, at which the handsome sum of £650 was realised. To this must be added £50, the proceeds of a concert which took place in the large hall at the Beckett's Park Hospital. It is evident that the College is spreading its roots deep and wide in Yorkshire.

It is satisfactory to learn that recruits have been coming in well during the last few weeks for the V.A.D. organisations. In particular, large numbers of women have arrived from Australia, Canada, and South Africa, singly and in parties of fifty, ready to be of use wherever they are wanted. There are still, however, vacancies for suitable candidates. The club-rooms at Devonshire House, with the garden canteen, have proved very serviceable to the Senior Women's War Service during the six months they have been open, and the advantages they offer ought to be more widely known in hospitals to which V.A.D. workers are attached. They are open free to all members of detachments on presentation of their uniform permit.

The great recruiting rally for Red Cross and Army nurses in the United States continues to make good progress. The country has been divided into thirteen sections, each of which has been asked to contribute a given number of nurses, and it is hoped by this means to secure at least 27,000 by January 1. By August 1 a total of 13,360 nurses had responded to the call and been assigned to duty. It is estimated that there are from 80,000 to 100,000 graduate nurses in the States from whom to draw. A few nursing aides are already at work, but it is not anticipated that it will be necessary to draw largely on amateur help.

We note that the Royal United Bath Hospital is admitting members of Voluntary Aid Detachments with two years' good record of consecutive work in a military hospital for three, instead of the usual four, years' training.

The Belfast Union Infirmary is distinguished among Irish Poor-Law institutions by the importance attached in it to the professional education of the nurses. Three medals, presented by different members of the Board of Guardians, were competed for by the probationers at their recent final examination, with the following result: *Gold Medal*, Nurse J. McAuley Harkness; *Silver Medal*, Nurse Rebecca Gleazer; *Bronze Medal*, Nurse Dorothy Magowan.

Bush nursing in Australia continues to progress in spite of the drain which the war is making on the Nursing Service of the Dominions. Last year's report shows that while new recruits for this branch of work are hard to obtain at the moment, the open, free life, even with its accompaniments of hard going, proves so attractive to those who are once enlisted that they stick to it, and even if lured away elsewhere come back in the end to the Centre where they have made friends for life. The finance is managed on a co-operative basis, every settler paying £1 a year for himself and his family below the age of eighteen. Over this age the charge is 5s. a year; and an extra charge is willingly paid for midwifery. School inspection is one of the nurses' most exacting duties, and includes the obligation of addressing the children on hygiene. Some Centres contain from six to ten schools, averaging 250 to 300 children each. The movement is thoroughly live, and has brought hope and help to hundreds of lonely settlers in the sparsely populated districts.

The Countess of Gosford is appealing for more helpers at the Central Work-rooms of the Red Cross at Burlington House, Piccadilly. The needs of the sick and wounded are daily increasing in all parts of the battle area, and more and more ladies are wanted to help. There are many nurses who could give half a day a week; many, debarred from active work, who could do a great deal more than this, some who could inspire others at present standing idle to do their share. The need can hardly be greater than at the present moment and the call is urgent to work *to-day*.

Two American ladies, Miss Anne Morgan and Mrs. Dike, who have devoted their energies to recreating homes in the devastated parts of France, have recently been awarded the Croix de Guerre, with palm, by the French authorities. In the spring of this year they had the mortification of seeing all their work undone, but the official report says of them both that they retreated step by step only after all civilians had been evacuated, and always under enemy fire. By their energy and courage they gave invaluable support to the military and civilian authorities during the evacuation, helping specially to protect the children, transport the wounded, and organise travelling canteens. Later on they helped to re-establish the inhabitants in their homes, and provided food and comforts for the wounded and for soldiers returning from the Front.

A useful pamphlet, "Food and How to Save It," price 3d., has been issued by H.M. Stationery Office, Imperial House, Kingsway, W.C. 2. The author is Mr. Edmund I. Spriggs, F.R.C.P., and the interest is divided pretty equally between the scientific and practical sides of the dietary. Admirable directions are given for keeping every kind of household plentifully supplied with nourishing food under existing conditions. The little book is a mine of information, and has many new ways to suggest of using the materials to hand.

